# The Nature, History, and Treatment of Childbed Fever

**Published:** 1846-01

**Authors:** 


ART. III.—*Das Kindbettfieber in nosologischer, geschichtlicher und therapeutischer Beziehung.* Von Dr. C. T. LITZMANN.—*Halle*, 1844.

*The Nature, History, and Treatment of Childbed Fever.* By Dr. LITZMANN. —*Halle*, 1844. 8vo, pp. 346.

PUERPERAL fever prevailed epidemically during the winter of 1840, in the Lying-in Hospital at Halle. It broke out in a cold and peculiarly ungenial season, and at a time when influenza was very prevalent, and continued from the end of December until the return of warmer weather at the beginning of the following April. Fourteen persons were attacked by it, eight of whom died, but only three cases occurred out of the hospital, and in each of them the influence of contagion was plainly traceable. In those cases also the patients died. The peritoneum, ovaries, and fallopian tubes were the chief seats of local mischief, and secondarily, the mucous membrane of the stomach and colon were frequently affected. The disease usually commenced on the evening of the second day after delivery, and an extraordinary rapidity of the pulse which beat 130 or 140 times in the minute, though continuing soft and small, was usually its first symptom. The lochial discharge was unaffected, but the secretion of milk was soon suspended, the abdomen was soft, and the pain experienced then at first dull, deep seated, and excited only by pressure. During the night the patients continued restless and feverish with heaviness of the head and uneasiness of the abdomen, but, towards morning the occurrence of a gentle perspiration relieved all the symptoms. Towards evening, however, there was a fresh exacerbation of the disease, the abdomen became distended, and in a short time extremely painful and the patients grew exceedingly restless and anxious. The constipated state of the bowels was suddenly succeeded by profuse watery diarrhea, attended with tenesmus, and pain in the course of the colon, and retching with vomiting of a bilious fluid. The secretion of milk ceased at so early a period after the commencement of the illness, that the child was never suckled for more than a very short time. In no instance, however, did the milk appear to exert any injurious influence upon them, and all the infants but one, were discharged from the hospital quite well.

Dr. Litzmann had the opportunity of observing this epidemic during its whole course, as he then held the appointment of assistant-physician to the hospital. We should not, however, have noticed his work, had it contained nothing more than an account, how good soever that account might have been, of a comparatively unimportant epidemic. But Dr. Litzmann has brought a truly German industry to bear upon the subject, and has compiled an extremely accurate history of every important epidemic of puerperal fever of which we have any record. This historical sketch occupies nearly two hundred pages of the work, and supplies a want long felt in obstetric literature. In another part of his treatise he mentions a fact of considerable interest in the history of the disease; namely, that peritoneal inflammation formed the basis, of nearly all the epidemics of puerperal fever that prevailed in the eighteenth century. It appears to have for the most part run its course in an uncomplicated form, and to have been comparatively seldom associated with inflammation of other parts of the sexual apparatus. In the present century, on the contrary, peritoneal inflammation seems to have characterized the epidemics only at their commencement and so long as the disease retained a sthenic character, and to have subsequently given way to other varieties. Closely connected with this is the fact that uterine phlebitis has been gradually increasing in frequency, and has constituted the grand characteristic of many of the fatal epidemics that have prevailed in the hospitals of Paris, London, and Vienna, during the past twenty years. The historical facts adduced by Dr. Litzmann show that this apparent change in the character of puerperal fever, cannot be by any means satisfactorily explained on the supposition that former epidemics were imperfectly observed; but that a real alteration in the *genius epidemicus* must have taken place. It would be an interesting task, but one on which we cannot now pretend to enter, to trace the revolutions which have taken place in the epidemic constitution within the past quarter of a century.

The reasons that have led most eminent recent observers to consider puerperal fever as a blood disease, are ably expounded by Dr. Litzmann who himself advocates that opinion. He does not adduce any new arguments on the subject, but relates the different observations and experiments of others with great precision. The account of the morbid anatomy and symptoms of the disease, the description of its various forms, and, indeed, the whole of the remainder of the work present the same proofs of sound and extensive learning, and great good sense which raise it far above the level of ordinary compilations, while the historical notices that it contains, and which might be vainly sought for elsewhere, will render it an invaluable aid to all teachers of midwifery who are masters of the German language.

